# Predictive value of preoperative erythrocyte electrophoresis exponent for acute deep vein thrombosis after total knee arthroplasty in patients with knee osteoarthritis

**DOI:** 10.1186/s13018-020-02020-x

**Published:** 2020-10-28

**Authors:** Dai Xiaoyu, Chen Kai, Huang Zhihui, Li Huan, Zhang Naidong, Ding Wenge

**Affiliations:** 1 Department of Orthopedic Surgery, The First People’s Hospital of Changzhou Affiliated to Soochow University, Juqian Road 185, Changzhou, 213000 Jiangsu People’s Republic of China; 2 Department of Orthopaedics, Hai’an People’s Hospital Affiliated to Nantong University, Zhongba Road 17, Hai’an, 226600 Nantong, Jiangsu People’s Republic of China

**Keywords:** Osteoarthritis, Knee, Erythrocyte electrophoresis, Total knee arthroplasty, Deep vein thrombosis

## Abstract

**Background:**

Hemorheological parameters have been confirmed to be related with deep vein thrombosis (DVT). This study is aimed to verify whether preoperative erythrocyte electrophoresis exponent was associated with postoperative deep vein thrombosis (DVT) risk after total knee arthroplasty (TKA) in patients with primary knee osteoarthritis (KOA).

**Methods:**

From March 2010 to May 2020, a total of 750 consecutive KOA patients who accepted unilateral TKA were enrolled. They were divided into DVT (176 patients) and non-DVT groups (574 patients) according to the examination results of the Doppler ultrasound of deep veins in both lower limbs on postoperative day 3. The Chi-square test, Student’s *t* test, and multivariate logistic regression analysis were performed to analyze the correlation of erythrocyte electrophoresis exponent and DVT risk in 2 groups. Receiver operating characteristic (ROC) analysis was used to assess predictive value of erythrocyte electrophoresis exponent for DVT.

**Results:**

A low erythrocyte electrophoresis exponent was a significant risk factor for DVT in patients with primary KOA (*p* < 0.05), especially in females when stratified by gender (*p* < 0.05).

**Conclusions:**

The findings suggest that lower erythrocyte electrophoresis before surgery may be independently associated with a higher post-surgery DVT risk in primary KOA patients. It is necessary to optimize prophylaxis strategies for DVT in these patients.

## Introduction

Osteoarthritis (OA) is the most prevalent form of degenerative arthritis and a major cause of pain and disability that preferentially affected the middle-aged and elderly people in global [1, 2]. Particularly, knee joint is the most common site of OA, and KOA is expected to be the fourth leading cause of disability in 2020 [2]. Acted as a kind of orthopedic surgery, TKA is evidently associated with the improvements in KOA patients’ life quality [3]. Venous thromboembolism (VTE), which comprises DVT and pulmonary embolism PE, is a major and potentially fatal complication following TKA [4]. Notably, DVT incidence after TKA ranged from about 18.1 to 48.6% without thromboprophylaxis in Asian patients [4]. Although the available guidelines [5] recommended that KOA patients should undergo routine thrombosis prevention after TKA, adverse complications of bleeding and deep inflammation cannot be ignored [4, 6]. For all this, it is of considerable significance to find some presurgical biomarkers which may partly predict postsurgical DVT risk that will highly contribute to a more targeted and integral thrombosis prophylaxis with less side effects.

Several lines of evidence have suggested that many recognized DVT risk factors like aging, obesity, hypertension, diabetes, smoking, dyslipidemia, as well as adverse dietary habits and other lifestyle factors were correlated with hemorheological changes such as increased erythrocyte aggregation, blood, or plasma viscosity and decreased erythrocyte deformability [7, 8]. Importantly, erythrocytes are thought to be incorporated into venous thrombi via specific interactions, culminating in the formation of an erythrocyte- and fibrin-rich venous thrombus [9]. It has been illustrated that erythrocytes are negatively charged mainly due to the sialic acid or N-acetylneuraminic acid on their surface and can move to the positive electrode in an electric field [10]. The changes of the blood internal environment caused by some specific factors can reduce the negative charge on erythrocytes’ surface, decrease the spacing of erythrocytes, and promote erythrocytes to aggregate into a rouleaux formation, thereby prolonging the electrophoresis time of erythrocytes and ultimately lead to the reduction of erythrocyte electrophoresis exponent [10]. Taken together, it followed that erythrocyte electrophoresis exponent might play an underlying role in the onset, development, and outcome of DVT. Considering a large number of KOA patients who ultimately need to undergo TKA, the present work is a bold attempt to find out the association of preoperative erythrocyte electrophoresis exponent and acute DVT risk after TKA.

## Methods

### Participants

This study was approved by the ethical committee of our hospital, and informed consents were obtained from all the patients. From March 2010 to May 2020, we reviewed the medical records of 1613 consecutive patients who had undergone TKA due to end-stage KOA. Exclusion criteria were (1) planned bilateral TKAs; (2) RA or other definite disease of immune system such as ankylosing spondylitis and systemic lupus erythematosus; (3) congenital or acquired coagulopathy and preoperative use of anticoagulant therapy; (4) hemorheological disease such as myeloma, hemophilia, Waldenström disease, and polycythemia vera; (5) immobilization of the lower extremities for more than 3 days before operation; (6) previous operations on the suffered leg within 12 months before total joint arthroplasty; (7) steroids or hormone therapy within the last 3 years; (8) blood transfusion within 12 months. Accordingly, a total of 750 patients met the criteria and were incorporated into this retrospective study.

The weight and height of each patient were measured to calculate the body mass index (BMI) value. Clinical information including the potential risk factors and medical histories were collected according to their self-report, which mainly consisted of age, gender, diabetes, hypertension, malignancy, cardiovascular events (i.e., diseases originate from atherothrombosis including myocardial infract, stable or unstable angina, or stoke), VTE events, steroids or estrogen use, smoking, surgery history, and blood transfusion history. All the information were recorded by the same doctor.

### Blood sampling and TKA

A day after admission, venous blood of each untreated patient was routinely sampled at 7 a.m. from a forearm vein with a 21 gauge “butterfly” needle (Becton Dickinson Medical Device Co., Ltd. Su Zhou) after overnight fasting (12–14 h). Each blood specimen was immediately sent and evaluated in the clinical laboratory of our hospital. Thereinto, blood for fibrinogen (FIB), prothrombin time (PT), activated partial thromboplastin time (APTT), thrombin time (TT), and D-dimer were tested with CA-7000 automatic blood coagulation analyzer (Sysmex Co., JPN). Platelet count and serum levels of triglycerides (TG), total cholesterol (TC), high density lipoprotein cholesterol (HDL-C), low density lipoprotein cholesterol (LDL-C), apolipoprotein A1 (apo A1), and apolipoprotein B (apo B) were tested by HITACHI 7600-20 fully biochemical autoanalyzer (HITACHI, JPN). Erythrocyte electrophoresis exponent was examined by LBY-N6C automatic hemorheology instrument (Precil Co., CHN).

All surgeries were performed under general anesthesia. All TKAs consisted of condylar-type and posterior cruciate substituting cemented arthroplasty via a knee midline skin incision and a deep medial parapatellar approach. All patients had identical DVT prophylaxis of low molecular weight heparin (LMWH) which was given at 10 h after surgery. Patients were encouraged to contract the gastrocnemius and hamstring muscle immediately. The early active mobilization started within 3 to 5 h after surgery. We routinely monitored the clinical symptoms and signs of acute DVT such as pain and tenderness of the limb, swelling, and erythema of the thigh or calf. Patients were instructed to stand up and start walking as soon as the drainage was removed in 48 h after definite good cement fixation or press-fit by X-ray.

### DVT diagnosis and treatment

All patients underwent routine ultrasound examination of bilateral lower extremity veins for DVT screening on the 3rd day following TKA. The diagnosis criterion for DVT was made according to Rabinov and Paulin in 1972 [11]. Proximal DVT was defined as thrombosis of the popliteal or more proximal veins with or without concomitant calf vein thrombosis. Distal DVT was defined as thrombosis affecting the calf veins. Patients with DVT on either side of the leg were assigned to the DVT group. DVT patients were all treated with LMWH, and ultrasound reexamination of both lower limbs were done every 3 days to observe the change and outcome of thrombosis. Proximal DVT patients were restricted from getting out of bed, and all DVT patients were discharged from the hospital when the thrombosis improved such as the reduction of thrombosis size and blood vessel recanalization. After discharge, anticoagulant therapy is still required for DVT patients for at least 3 months according to the guidelines.

### Statistical analysis

The χ^2^ test was used to compare differences in categorical variables, and Student’s *t* test was adopted to make a comparison of numerical variables. Age, gender, and BMI and all other preoperative confounding factors were adjusted for multivariate logistic regression analysis. ROC analysis was also made to assess predictive value of erythrocyte electrophoresis exponent for DVT. All data was analyzed by SPSS 19.0 (IBM SPSS, Chicago, America). A *p* value of less than 0.05 was considered statistically significant.

## Results

### Study population

In Table 1, 176 patients were diagnosed as DVT (4 proximal DVTs, 164 distal DVTs, and 8 mixed DVTs), and the total incidence of postoperative DVT was 23.5% (176/750). No one dropped out during hospitalization. Clinical characteristics of the patients with and without postoperative DVT are showed in Table 2. Thereinto, the incidence rate of DVT was 16.7% (24/144) in males and 25.1% (152/606) in females, respectively. This difference between men and women was statistically significant with a higher incidence in females (*p* = 0.032). The smoking rate was lower in DVT patients (2.2%, 4/176) than that in non-DVT patients (42/574, 7.3%) (*p* = 0.015). In addition, no significant differences were seen in the distribution of diabetes, hypertension, malignancy, cardiovascular events, VTE events, steroids or estrogens use, surgery history, and blood transfusion history between DVT and non-DVT groups (*p* > 0.05). In Table 2, a parallel mean age and BMI were observed between two groups, even after stratified by gender (*p* > 0.05). Still, there were no significant differences in TG, HDL-C, apo A1, apo B, FIB and D-dimmer levels, platelet count, PT, APTT, and TT (*p* > 0.05). It was worth noting that DVT group had the significantly higher TC (*p* = 0.010) and LDL-C levels (*p* = 0.018) than non-DVT group.

**Table 1:** The locations of DVT

Locations	no.
**Distal DVT**	164
Anterior tibial vein	2
Posterior tibial vein	6
Peroneal vein	2
Muscular veins	96
Muscular + peroneal veins	22
Muscular + posterior tibial veins	6
Muscular + peroneal + posterior tibial veins	22
Muscular + peroneal + anterior tibial + posterior tibial veins	8
**Proximal DVT**	4
Femoral vein	2
Popliteal vein	2
**Mixed DVT**	8
Peroneal + popliteal veins	2
Muscular + peroneal + posterior tibial + popliteal veins	6

*DVT* deep vein thrombosis, *no.* number

**Table 2:** Clinical characteristics of DVT and non-DVT patients

Clinical characteristics	DVT patients	Non-DVT patients	*p* value
Patients, no.	176	574	-
Mean age, years (SD)	67.18 (7.435)	66.62 (8.185)	0.416
Females, years (SD)	67.04 (7.678)	65.86 (8.393)	0.126
Males, years (SD)	68.08 (5.710)	69.50 (6.624)	0.330
Male gender (males/females, no.)	24/152	120/454	**0.032**^**a**^
Mean BMI, kg/m^2^ (SD)	26.45 (3.974)	26.05 (3.872)	0.241
Females, kg/m^2^ (SD)	26.56 (4.110)	26.21 (3.933)	0.341
Males, kg/m^2^ (SD)	25.74 (2.942)	25.48 (3.591)	0.744
Diabetes mellitus (with/without, no.)	30/146	86/488	0.508^a^
Hypertension (with/without, no.)	88/88	290/284	0.903^a^
Malignancy (with/without, no.)	8/168	10/564	0.065^b^
Cardiovascular events (with/without, no.)	12/164	62/512	0.121^a^
VTE events (with/without, no.)	12/164	44/530	0.708^a^
Steroid or estrogen use (with/without, no.)	8/168	48/526	0.092^a^
Smoking (with/without, no.)	4/172	42/532	**0.015**^**a**^
Surgery history (with/without, no.)	34/142	126/448	0.456^a^
Blood transfusion history (with/without, no.)	26/150	86/488	0.946^a^
TG, mmol/L (SD)	1.41 (0.644)	1.50 (0.981)	0.213
TC, mmol/L (SD)	4.81 (0.832)	4.62 (0.890)	**0.010**
HDL-C, mmol/L (SD)	1.35 (0.342)	1.31 (0.330)	0.096
LDL-C, mmol/L (SD)	2.57 (0.665)	2.44 (0.625)	**0.018**
Apo A1	1.24 (0.211)	1.21 (0.219)	0.086
Apo B	0.95 (0.207)	0.93 (0.213)	0.322
Platelet count, 10^12^/L (SD)	203 11 (61.265)	209.16 (71.910)	0.314
FIB, g/L (SD)	3.18 (0.745)	3.26 (0.794)	0.218
D-dimer, mg/L (SD)	0.65 (1.270)	0.74 (1.266)	0.394
PT, s (SD)	11.31 (0.736)	11.20 (1.047)	0.183
APTT, s (SD)	25.15 (3.136)	25.48 (4.131)	0.327
TT, s (SD)	17.69 (1.730)	17.40 (1.736)	0.052

*no*. number, *BMI* body mass index, *SD* standard deviation, *VTE* venous thromboembolism, *TG* triglycerides, *TC* total cholesterol, *HDL-C* high density lipoprotein cholesterol, *LDL-C* low density lipoprotein cholesterol, *apo A1* apolipoprotein A1, *apo B* apolipoprotein, *FIB* fibrinogen, *PT* prothrombin time, *APTT* activated partial thromboplastin time, *TT* thrombin time

^a^Pearson Chi-square test

^b^Continuous correction test

### Comparison of erythrocyte electrophoresis exponent

In Table 3, the significant difference of erythrocyte electrophoresis exponent between two groups was showed in all the patients (*p* = 0.003), especially in female patients when stratified by gender (*p* = 0.000). In the multivariate analysis (Table 3), it could be found that a low erythrocyte electrophoresis exponent was a significant risk factor for postoperative DVT in both all the patients (OR = 0.606, 95% CI = 0.436–0.841, *p* = 0.003) and female patients (OR = 0.568, 95% CI = 0.399–0.810, *p* = 0.002) after stratified by gender with adjustment for age, BMI, serum lipids, coagulation indexes, and all the other confounding factors in this study. In males, there existed no statistical differences in erythrocyte electrophoresis exponent (*p* > 0.05) (Table 3).

**Table 3:** Comparisons of erythrocyte electrophoresis exponent

Erythrocyte electrophoresis exponent	All	*p* value	Females	*p* value	Males	*p* value
**Mean (SD)**						
DVT patients	4.75 (0.594)	0.003	4.76 (0.617)	0.000	4.64 (0.575)	0.967
Non-DVT patients	4.91 (0.664)		4.99 (0.667)		4.64 (0.419)	
**Multivariate analysis**	OR^a^ (95% CI)	0.003	OR^b^ (95% CI)	0.002	OR^b^ (95% CI)	0.298
	0.606 (0.436–0.841)		0.568 (0.399–0.810)		0.314 (0.035-2.776)	

*SD* standard deviation, *DVT* deep vein thrombosis, *OR* odds ratio, *CI* confidence interval

^a^Odds ratio adjusted for matching factors of gender, age, BMI, and all the confounding factors in this study

^b^Odds ratio adjusted for matching factors of age, BMI, and all the confounding factors in this study after stratified by gender

### ROC analysis of erythrocyte electrophoresis exponent

**Fig. 1:**
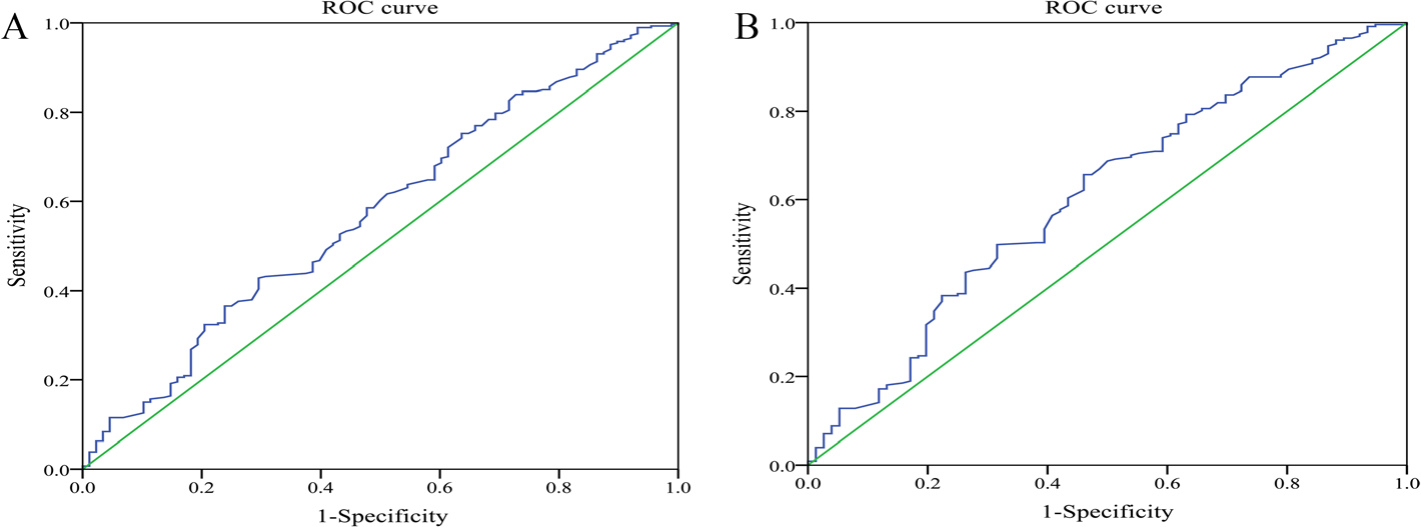
ROC analysis of erythrocyte electrophoresis exponent to predict postoperative DVT in all patients (**a**) and female patients (**b**)

In the ROC analysis, the AUC of erythrocyte electrophoresis exponent was 0.574 in all the patients (95% CI = 0.525–0.623, *P* = 0.000) and 0.606 in female patients (95% CI = 0.554–0.659; *P* = 0.000) (Fig. 1).

## Discussion

For the first time, this study retrospectively investigated the underlying effects of preoperative erythrocyte electrophoresis exponent on postoperative acute DVT after TKA in primary KOA patients. Compared with non-DVT group, a lower erythrocyte electrophoresis exponent was associated with an increased risk of DVT, especially in females. Although the exact mechanisms linking erythrocyte electrophoresis and DVT are not yet unequivocally established, our findings can result in important insights about erythrocyte electrophoresis in DVT formation.

Intrinsically, VTE is initiated by inflammation and blood stasis leading to the generation of thrombi rich in fibrin and erythrocytes [9]. Erythrocytes contribute to VTE mainly due to a rise in blood viscosity, marginate platelets and fibrin deposition, an increase in erythrocyte aggregation (EA), a decrease in erythrocyte deformability, or its self-generated complex effects on blood clot structure and stability [9, 12, 13]. It can be proposed that erythrocytes are not only major components of venous thrombi and determinants of VTE risk, but also actively involved in VTE pathophysiology [9]. From the perspective of hemorheology, erythrocyte has a negative surface charge (zeta potential) and can be examined by erythrocyte electrophoresis mobility which can keep the cells in an appropriate distance [14, 15]. The vessel wall also carries negative charges which provide repulsion to blood cells so as to prevent erythrocytes from adhering to the intima and prevent vascular thrombosis [15]. As we know, the lower electrophoresis exponent means the reduced surface charge of erythrocytes and may indicate the more tendency of EA. EA is mainly manifested by rouleaux (linear arrays of stacked cells) or two- and three-dimensional aggregates with blood stasis in an osmotic gradient mechanism, which is thought to be reversible and shear dependent [16, 17]. Noteworthily, several lines of evidence have suggested that erythrocyte hyperaggregation was a kind of classic prothrombotic abnormalities in first time and recurrent DVT patients [18, 19]. The aggregates of erythrocytes can potentially increase blood viscosity and hydrodynamic resistance of larger vessels such as lower extremity veins [17, 18]. Taken together, it is tempting to speculate that the alterations of erythrocyte electrophoresis can lead to the changes in EA and thus play a potential role in the pathogenesis of DVT.

Previously, Saha et al. have indicated that the possible damage to the vessel wall during the operation, the venous stasis caused by long-term bed rest, and blood hypercoagulability after surgery are the 3 main reasons for the formation of DVT [20]. Combined with the outcomes of the present study, it is plausible that erythrocytes in patients with a low erythrocyte electrophoresis exponent before surgery are more likely to be affected by multiple factors during surgery which may cause a large increase of EA that ultimately have an underlying impact on DVT. However, what cannot be ignored is that systemic hemorheological alterations may be not comparable to those in local areas where minimum disturbances can be more relevant to DVT [7, 18]. Although our findings partly provided insight into the continued work in the field of the prognostic value of erythrocyte electrophoresis, confirming the pathogenic effects of locally altered erythrocyte electrophoresis in the development of DVT is quite a necessity. Until now, there are no any other clinical studies on the correlation between erythrocyte electrophoresis and DVT. Different confounding factors used for adjustment, testing time points for erythrocyte electrophoresis, and study populations should be taken into account in subsequent researches for a more accurate and comprehensive result. In addition, by a subgroup analysis, the significant association was only found in female patients might be due to a higher KOA incidence in females than that in males.

DVT after total joint arthroplasty has many well-identified clinically associated or risk factors such as increasing age, previous VTE, malignancy, obesity, diabetes, hyperlipidemia, cardiovascular disease, and immobility [5]. Over the past several decades, OA has been increasingly recognized as a low­grade inflammatory condition with an elevation in systemic inflammatory mediators such as interleukin-1 (IL­1), IL­6, and tumor necrosis factor (TNF) [1]. Notably, the elevated risk of VTE is deemed to be correlated with increased expression of IL-6, TNF-α, and some other inflammatory biomarkers [21, 22]. Accordingly, the chronic low-grade inflammatory status caused by KOA may be susceptible to multiple factors during TKA surgery and further accelerate the occurrence of DVT by imperceptibly affecting the patient’s erythrocyte electrophoresis and EA. To our best knowledge, the specific link and interaction between inflammation and erythrocyte electrophoresis still remains elusive for researchers; therefore, further researches should be carried on.

There existed both some strengths and limitations in this study. We adjusted the confounding factors such as age, BMI, hypertension, diabetes, serum lipids, and indicators related to coagulation function which may potentially affect hemorheological parameters. Also, the sample size is relatively large with a long study period, and there is reason to believe that the findings are of certain reliability and practicality. Notwithstanding this, we did not conduct a dynamic monitoring on erythrocyte electrophoresis after TKA to do a further analysis. Simultaneously, in light of the high proportion of patients with distal DVT (164/176, 93.2%), we fail to further analyze the correlation of the erythrocyte electrophoresis exponent and DVT locations.

## Conclusion

For primary KOA patients treated with TKA, low erythrocyte electrophoresis exponent is evidently associated with postoperative DVT. Definitely, the results may highlight the significance of hemorheological analysis in DVT etiology and improving prophylaxis strategies.

## Data Availability

The datasets used and/or analyzed during the current study are available from the corresponding author on reasonable request.

## Abbreviations

KOA: Knee osteoarthritis
OA: Osteoarthritis
DVT: Deep vein thrombosis
VTE: Venous thromboembolism
BMI: Body mass index
TKA: Total knee arthroplasty
TG: Triglycerides
TC: Total cholesterol
HDL-C: High density lipoprotein cholesterol
LDL-C: Low density lipoprotein cholesterol
apo A1: Apolipoprotein A1
apo B: Apolipoprotein
FIB: Fibrinogen
PT: Prothrombin time
APTT: Activated partial thromboplastin time
TT: Thrombin time
EA: Erythrocyte aggregation
IL: Interleukin
TNF: Tumor necrosis factor
OR: Odds ratio
CI: Confidence interval
SD: Standard deviation
